# Longitudinal Cohort Study of Gender Affirmation and HIV-Related Health in Transgender and Gender Diverse Adults: The LEGACY Project Protocol

**DOI:** 10.2196/24198

**Published:** 2021-03-01

**Authors:** Sari L Reisner, Madeline B Deutsch, Kenneth H Mayer, Jennifer Potter, Alex Gonzalez, Alex S Keuroghlian, Jaclyn MW Hughto, Juwan Campbell, Andrew Asquith, Dana J Pardee, David R Pletta, Asa Radix

**Affiliations:** 1 Division of Endocrinology, Diabetes and Hypertension Brigham and Women’s Hospital Boston, MA United States; 2 Harvard Medical School Boston, MA United States; 3 University of California San Francisco San Francisco, CA United States; 4 Harvard TH Chan School of Public Health Boston, MA United States; 5 The Fenway Institute Boston, MA United States; 6 Beth Israel Deaconess Medical Center Boston, MA United States; 7 Massachusetts General Hospital Boston, MA United States; 8 Brown University School of Public Health Providence, RI United States; 9 Center for Health Promotion and Health Equity Brown University Providence, RI United States; 10 Callen-Lorde Community Health Center New York, NY United States

**Keywords:** cohort studies, transgender persons

## Abstract

**Background:**

Transgender and gender diverse (TGD) adults in the United States experience health disparities, especially in HIV infection. Medical gender affirmation (eg, hormone therapy and gender-affirming surgeries) is known to be medically necessary and to improve some health conditions. To our knowledge, however, no studies have assessed the effects of gender-affirming medical care on HIV-related outcomes.

**Objective:**

This study aims to evaluate the effects of medical gender affirmation on HIV-related outcomes among TGD primary care patients. Secondary objectives include characterizing mental health, quality of life, and unmet medical gender affirmation needs.

**Methods:**

LEGACY is a longitudinal, multisite, clinic-based cohort of adult TGD primary care patients from two federally qualified community health centers in the United States: Fenway Health in Boston, and Callen-Lorde Community Health Center in New York. Eligible adult TGD patients contribute electronic health record data to the LEGACY research data warehouse (RDW). Patients are also offered the option to participate in patient-reported surveys for 1 year of follow-up (baseline, 6-month, and 12-month assessments) with optional HIV and sexually transmitted infection (STI) testing. Biobehavioral data from the RDW, surveys, and biospecimen collection are linked. HIV-related clinical outcomes include pre-exposure prophylaxis uptake (patients without HIV), viral suppression (patients with HIV), and anogenital STI diagnoses (all patients). Medical gender affirmation includes hormones, surgeries, and nonhormonal and nonsurgical interventions (eg, voice therapy).

**Results:**

The contract began in April 2018. The cohort design was informed by focus groups with TGD patients (n=28) conducted between August-October 2018 and in collaboration with a community advisory board, scientific advisory board, and site-specific research support coalitions. Prospective cohort enrollment began in February 2019, with enrollment expected to continue through August 2020. As of April 2020, 7821 patients are enrolled in the LEGACY RDW and 1756 have completed a baseline survey. Participants have a median age of 29 years (IQR 11; range 18-82). More than one-third (39.7%) are racial or ethnic minorities (1070/7821, 13.68% Black; 475/7821, 6.07% multiracial; 439/7821, 5.61% Asian or Pacific Islander; 1120/7821, 14.32% other or missing) and 14.73% (1152/7821) are Hispanic or Latinx. By gender identity, participants identify as 33.79% (2643/7821) male, 37.07% (2900/7821) female, 21.74% (1700/7821) nonbinary, and 7.39% (578/7821) are unsure or have missing data. Approximately half (52.0%) of the cohort was assigned female sex at birth, and 5.4% (421/7821) are living with HIV infection.

**Conclusions:**

LEGACY is an unprecedented opportunity to evaluate the impact of medical gender affirmation on HIV-related health. The study uses a comprehensive research methodology linking TGD patient biobehavioral longitudinal data from multiple sources. Patient-centeredness and scientific rigor are assured through the ongoing engagement of TGD communities, clinicians, scientists, and site clinical staff undergirded by epidemiological methodology. Findings will inform evidence-based clinical care for TGD patients, including optimal interventions to improve HIV-related outcomes.

**International Registered Report Identifier (IRRID):**

DERR1-10.2196/24198

## Introduction

### Background

In the United States, transgender and gender diverse (TGD) adults experience disparities in HIV-related outcomes, particularly TGD women who have an estimated 21.7% laboratory-confirmed HIV prevalence (meta-analysis), a 34.2-fold increased odds relative to the US general population [1]. Black and Latinx TGD people are particularly hard-hit by the HIV epidemic [2,3]. TGD men are also at risk for HIV acquisition and transmission, particularly TGD men who are gay, bisexual, or have sex with other men [2,4-7]. Data are lacking about the HIV epidemic in nonbinary TGD people [3,4]. TGD people are a priority population for HIV biobehavioral prevention and care efforts [8]. HIV testing is vital in identifying new HIV infections and linking TGD individuals to antiretroviral treatment [8]. Pre-exposure prophylaxis (PrEP) has shown efficacy in reducing HIV incidence in TGD people without HIV, offering options for clinically delivered prevention interventions [9]. There is substantial variability in viral suppression rates among TGD people with HIV (eg, 50%-81%) [10-12]. For TGD individuals living with HIV, viral suppression is an important clinical outcome to reduce morbidity and mortality. It is also key to public health strategies such as U=U (undetectable=untransmittable) aimed at curbing onward transmission of HIV to sexual partners [8]. Multiple individual (eg, demographic), interpersonal (eg, violence), and structural (eg, stigma) factors increase HIV acquisition or transmission risks in TGD people, manifested by decreased rates of PrEP uptake [13] and viral suppression [4,10]. These risk factors are driven by and associated with barriers limiting access to gender-affirming HIV prevention, care, and health services [14-16].

TGD-related HIV disparities are situated alongside adverse mental health conditions (eg, depression, anxiety, and posttraumatic stress disorder), poor psychological functioning, and low health-related quality of life [16-23]. For example, the rates of suicidality among TGD people are devastatingly high, as evidenced by a US national survey of more than 27,000 TGD adults, which found that 40% reported one or more suicide attempts in their lifetime [24]. Behavioral health conditions adversely affect HIV prevention and care outcomes in TGD people [4,16]. In a 3-year prospective study of TGD women in New York, depressive distress predicted incident HIV or sexually transmitted infection (STI) [25]. In young TGD women living with HIV, those meeting the clinical criteria for depression had an increased probability of having a detectable viral load than those without depression [26]. Histories of psychosocial distress in TGD men are associated with self-reported STI diagnoses, a higher number of sexual partners, and condomless anal or vaginal sex [27,28]. Addressing TGD people’s mental health needs and improving psychological functioning are vital components of HIV prevention and treatment interventions [16,29].

Medical gender affirmation therapies—hormones and surgical interventions—are medically necessary treatments shown to improve psychological functioning and quality of life for TGD adults [17,30-37]. It is unknown whether these interventions improve HIV-related outcomes over time in adult TGD patients with diverse gender identities [38]. This is because studies providing the best evidence of medical gender affirmation’s clinical effectiveness do not examine outcomes along the HIV prevention continuum (eg, PrEP uptake and adherence) and the HIV care continuum (eg, viral suppression). Integrating medical gender affirmation with HIV prevention and care services may improve HIV-related outcomes for TGD people [29]. Clinical data on barriers and facilitators of medical gender affirmation and unmet needs of TGD people are also lacking. Studies characterizing medical gender affirmation in TGD people by age, race, ethnicity, gender identity, and HIV serostatus are lacking but are paramount to guide health care services and provide patient-centered clinical care [39]. This study will fill these gaps in evidence.

### Objectives

The specific aims of this study are to (1) evaluate whether medical gender affirmation improves HIV prevention and care outcomes over 12 months of follow-up, accounting for individual, interpersonal, and structural factors; (2) examine whether medical gender affirmation predicts 12-month prospective improvements in psychological functioning and health-related quality of life (HRQL) in TGD patients initiating hormone therapy adjusting for individual, interpersonal, and structural factors; and (3) characterize patient satisfaction with medical gender affirmation received, unmet needs and future desires, and barriers and facilitators of medical gender affirmation by age, race, ethnicity, gender identity, and HIV serostatus.

### Rationale

Lack of requisite knowledge concerning medical gender affirmation and HIV prevention and care outcomes impede the design, implementation, evaluation, and funding of health care and service delivery models that may reduce HIV disparities for TGD people. Patient-centered care must address and foreground those health issues important to TGD patients. Medical gender affirmation, such as access to and initiation of hormones, is a critical health concern for many TGD patients [40,41]. The delivery of medical gender affirmation in primary care may promote engagement with HIV prevention and care services for TGD people and improve psychological functioning and quality of life. Knowledge obtained from this first-of-its-kind study will inform the delivery of health care responsive to the specific concerns of TGD communities and lead to informed HIV-response efforts for TGD patients, a vulnerable health disparities population for whom clinical effectiveness research is urgently needed. This project will have a national impact on delivering medical gender affirmation in primary care and on intervention models to address HIV and related health disparities for TGD patients.

## Methods

### Overview

The LEGACY study is being conducted at The Fenway Institute at Fenway Health in Boston, Massachusetts, and Callen-Lorde Community Health Center in New York, New York, 2 federally qualified community health centers with long histories of providing culturally responsive and affirming health care for sexual and gender minority people, including TGD adults [42]. Fenway Health and Callen-Lorde were selected as sites because each has a large medical panel of unduplicated adult TGD patients. The Brigham and Women’s Hospital is the prime administrative site. All study procedures are approved by the Fenway Health Institutional Review Board (IRB; FWA00000145), which provides single IRB review for this study. All study data are managed by the Fenway Health data informatics team (NCT03595956).

### Conceptual Framework: A Biopsychosocial Model of Gender Affirmation and Hierarchy of Needs

**Figure 1:**
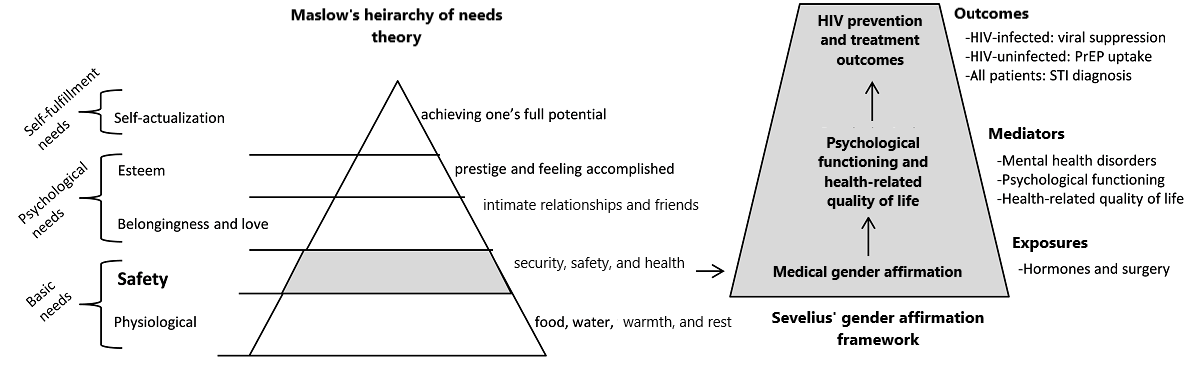
LEGACY cohort: the biopsychosocial model of gender affirmation and the hierarchy of needs in HIV prevention and treatment outcomes among transgender and gender diverse patients. PrEP: pre-exposure prophylaxis; STI: sexually transmitted infection.

This study applies a biopsychosocial model wherein biological, psychological, and social factors are expected to shape health outcomes [43,44]. Within this model, we draw on 2 conceptual frameworks (Figure 1). First, the Model of Gender Affirmation by Sevelius [45] conceptualizes that being affirmed in one’s gender influences psychological functioning and health behaviors (eg, HIV risk behaviors) for TGD people. A high need for gender affirmation and low access to gender affirmation are theorized to fuel poor HIV-related outcomes. Within a biopsychosocial model, it is also possible that hormonal and other system changes accompanying medical gender affirmation exert biological or clinical influences on psychosocial functioning. Second, the hierarchy of needs theory by Maslow [46,47] describes the pattern of motivations that humans generally move through to meet their needs. The theory suggests that at any given time, a certain need *dominates*. The most basic needs (ie, security, safety, and health) must be met before the individual will strongly desire (or focus motivation on) the higher-level needs (ie, belongingness and love, esteem, and self-actualization). Integrating the gender affirmation and hierarchy of needs frameworks, gender affirmation—social, psychological, medical, and legal [48]—takes precedence in the hierarchy of needs for TGD people, given that it pertains to security, safety, and health. Medical gender affirmation, for those TGD people who seek it, is a *dominating* health need that, once met, facilitates TGD patients’ abilities to address other health issues, such as HIV prevention and treatment. In this study, medical gender affirmation (exposure) is the hypothesized driving factor in improving TGD patients’ psychological functioning and HRQL (mediators), thereby increasing TGD individuals’ capacity to become engaged in HIV prevention and care (outcomes).

### Formative Research: Patient Focus Groups

Formative in-person focus groups were conducted with TGD patients to inform the assembly of and data capture for the cohort. In total, 2 focus groups were conducted at Fenway Health and 2 were conducted at Callen-Lorde. Each group was facilitated by 2 TGD staff members using a semistructured interview guide to gather input on study activities and procedures. A total of 28 people participated in the focus groups. Focus groups were transcribed verbatim, and transcripts were thematically coded by 2 independent analysts using a constant comparative method [49].

### Community and Scientific Advisory Boards

A community advisory board (CAB) and a scientific advisory board (SAB) are actively engaged with the research team and provide input on all aspects of the study, including feasibility and acceptability of study procedures. The CAB comprises 7 individuals who identify as TGD or nonbinary people and who advise on keeping the study procedures community centered. The SAB comprises 7 individuals who are researchers and/or medical providers with expertise in transgender health and research methodologies. Each board meets at least twice per year to monitor study progress, troubleshoot challenges, and ensure the achievement of study aims. The SAB also acts as the data safety monitoring board for the study. Members are compensated for their time.

### Research Support Coalitions

At each study site, staff engagement is ensured through a research support coalition (RSC). The RSC is in place to represent the voice of staff from within partnering organizations. The RSC is separate from the CAB and SAB so that organizational personnel have the space to bring a staff perspective to project implementation, including feedback on proposed and implemented study activities. The RSC comprises 4 to 6 professional staff members from each partnering site. It includes administrators, clinicians, nurses, HIV prevention staff, and other support staff (eg, peer health navigators and case managers).

### Study Design

This longitudinal study comprised a multisite clinic-based cohort of adult TGD patients from Fenway Health and Callen-Lorde. Eligibility criteria for the LEGACY cohort was as follows: (1) aged 18 years or older (verified in the electronic health record [EHR]), (2) having a gender identity different from their sex assigned at birth (verified via a two-step method cross-categorizing natal sex and gender identity reported on patient registration and/or ICD-10 code of F64.0-9) [50,51], (3) being a current or new primary care patient at Fenway Health or Callen-Lorde (defined as those who had a medical visit within the past 12 months), and (4) having a signed patient consent form on file and no research exclusion documented in their patient chart. Patients’ biobehavioral data were collected from multiple sources (Table 1).

**Table 1:** LEGACY cohort: data sources.

Biobehavioral data collected				Patient		EHR^a^		Biomarker
**Primary outcomes: HIVPC^b^ and HIVCC^c^**								
	HIV-infected patients: viral suppression			—^d^		+^e^		●^f^
	HIV-uninfected patients: PrEP^g^ uptake			—		+		●
	All patients: STI^h^ diagnosis (chlamydia and gonorrhea)			X^i^		+		●
**Exploratory outcome: HIVPC and HIVCC**								
	HIV-uninfected patients: HIV incidence			—		+		●
**Descriptive variables: HIVPC and HIVCC**								
	**HIV-infected patients**							
		Initiation of ART^j^ and adherence to ART	X		+		—	
		Retention in care	—		+		—	
		CD4 count	—		+		●	
		History of opportunistic infections	—		+		—	
	**HIV-uninfected patients**							
		PrEP indication	X		+		—	
		PrEP adherence	X		+		—	
	**All patients**							
		HIV transmission risk behaviors	X		+		—	
**Exposures: medical gender affirmation**								
	**Primary objective exposure**							
		Hormones and surgery	X		+		—	
	**Subjective exposure**							
		Patient satisfaction	X		—		—	
**Descriptive variables**								
	Hormones: regimens and experiences			X		+		●
	Surgery types and experiences			X		+		—
	Street hormones and silicone use			X		+		—
	Anatomy inventory			—		+		—
**Mediators: mental health disorders, psychological distress, and quality of life**								
	Mental health and psychiatric diagnoses			X		+		—
	Psychological distress			X		+		—
	Health-related quality of life			X		—		—
**Covariates and confounders: individual, interpersonal, and structural**								
	**Individual**							
		Demographics and TGD^k^ history	X		+		—	
		Mental health care and medication utilization	X		+		●	
		Substance use behavior or disorder	X		+		—	
	**Interpersonal**							
		Transgender integration or adaptation	X		—		—	
		Violence victimization	X		+		—	
		Gender of sexual partners	X		+		—	
	**Structural**							
		Sex work, housing, and incarceration or jail	X		+		—	
		Stigma and discrimination	X		—		—	

^a^EHR: electronic health record.

^b^HIVPC: HIV prevention continuum.

^c^HIVCC: HIV care continuum.

^d^Data not collected from that source.

^e^Electronic health record data every 3 months.

^f^Biomarker or laboratory data.

^g^PrEP: pre-exposure prophylaxis.

^h^STI: sexually transmitted infection.

^i^Patient self-reported survey every 6 months.

^j^ART: antiretroviral therapy.

^k^TGD: transgender and gender diverse.

### Study Recruitment and Cohort Enrollment Procedures

The IRB granted a waiver of written consent to allow automatic enrollment of all existing and new TGD adult patients at Fenway Health and Callen-Lorde, who meet the study’s eligibility criteria, into the LEGACY research data warehouse (RDW). Deidentified EHRs data (eg, provider-documented diagnoses, biomarker and laboratory data, and pharmacy records) and computerized self-administered patient-reported outcomes (PRO) captured as part of routine care (eg, screening for smoking, depression, and violence) are extracted from the EHR every 6 months. All patients identified as eligible and enrolled in the LEGACY RDW are approached, either in-person at clinic sites with provider permission or via secure email, and asked to complete an additional LEGACY survey at 3 time points over 12 months (baseline, 6 months, and 12 months) and complete optional HIV and STI testing as part of their routine patient care. For HIV and STI testing, a trained phlebotomist at a partner’s lab collected blood for HIV-1/2 antigen and antibodies (fourth generation; >99.7% sensitivity and 100% specificity) and for syphilis (rapid plasma reagin and treponema pallidium particle agglutination confirmatory) testing. Urine, vaginal, and anorectal swabs will be provider- or self-collected (depending on participant preference) to test for *Neisseria gonorrhoeae* and *Chlamydia trachomatis* via the APTIMA COMBO 2 Assay (Gen-Probe; >95.2% sensitivity and >96.8% specificity). Participants must be able to read and understand English or Spanish and be willing and able to provide informed consent to participate in the additional survey. Initial eligibility is assessed via the EHR; patients are asked to verify their eligibility before consenting to the survey.

The electronic informed consent form (eICF) for the survey is programmed into REDCap (Research Electronic Data Capture) and is the second form of the electronic survey, preceded only by an eligibility confirmation form. The eICF describes and addresses all study procedures, including confidentiality and privacy, information about potential risks, discomforts and benefits of participation, and information regarding members of the research team to contact for further questions. It also states that participation is voluntary, that participants may decide not to take part or withdraw from the study at any time without penalty or loss of any benefits to which they might otherwise be entitled, and that study participation is in no way related to being able to access or continue receiving care or services at Fenway Health or Callen-Lorde. Participants are provided with contact information for study staff and are encouraged to call or speak with a staff member if they have any questions before consenting. If a participant agrees to join the study voluntarily, they are asked to consent to the following by checking the applicable boxes: (1) the survey, (2) optional HIV testing, and (3) optional STI testing. Participants who do not consent to optional HIV and/or STI testing are still provided the option to complete the survey.

Staff at both sites aim for patients’ consent to LEGACY RDW for survey administration while they are onsite for medical visits. Patients who are not reached this way and are instead contacted via secure email receive a message with information regarding the study and a unique survey link to a screener and consent form. Only eligible patients who provide informed consent are redirected to the survey questions. Survey links expire 14 days after a patient’s consent to participate during a medical visit. For patients who provide consent via their web-based unique survey link, the survey link expires 14 days after the secure email was sent. Patients are given the option to begin surveys at their medical visits and continue them remotely on the web should they be unable to complete the survey during their visit; however, surveys must be completed before their 2-week expiration date. If a survey is incomplete, survey progress is saved automatically during a patient’s visit, and a unique survey link is emailed to them to bring them to the last saved point in their survey. In addition, patients have the option of saving their progress on all remote surveys and continuing where they left off at their convenience; however, all surveys must be completed before their 2-week expiration date.

Patients enrolled in the LEGACY RDW or who complete the additional brief surveys integrated with routine patient care are not individually compensated. Patients who complete the surveys have the option to be entered into a raffle to win an Amazon gift card. At the end of the survey, patients are asked to indicate if they consent to be contacted either via the phone number or email (or both) listed on their patient record for the raffle. At each site, 2 winners are selected per month per assessment point. For example, of those who complete a baseline survey in the month of February, 2 are randomly selected from each site for a gift card. As surveys are completed, they are assigned a consecutive number in REDCap; a web-based random number generator is used to randomly select a number from the list of completed surveys within the specified month for the raffle. The winner is contacted via their preferred method and given 1 week to respond and/or claim their gift card.

### Patient Self-Reported Outcome Measures

The PRO measures in the LEGACY surveys are aligned with the study aims. Wherever possible, validated self-report measures from previous TGD research are asked to ensure cultural appropriacy and comparability across studies. Measures have been drawn from probability and nonprobability sample studies, including the US Transgender Population Health Survey [52], National Transgender Discrimination Survey [53], the 2015 US Transgender Survey [24], LITE Cohort [54], Project LifeSkills [55], Project VOICE [20,56], and TransMasculine Sexual Health Study [57].

Sociodemographic factors such as age, gender identity, sex assigned at birth, sexual orientation, racial or ethnic identity, employment, education, and income are queried. Sexual health measures include STI screening history and diagnoses [57], HIV testing history [58], HIV care cascade engagement for patients with HIV (antiretroviral therapy initiation and adherence using the Visual Analogue Scale) [59], HIV prevention cascade engagement for patients without HIV (PrEP indication, awareness, uptake, adherence, persistence, and side effects) [54], HIV transmission risk behaviors, and sexual partnerships including condomless sex and gender of sexual partners [60]. Medical gender affirmation assessment includes hormone use (age of initiation and access, regimens, side effects and experiences, and patient satisfaction), surgical procedures (current uptake, future desires for procedures, experiences and medical complications, and patient satisfaction), and medical gender affirmation outside of medical contexts (street hormones and silicone use).

Assessment of psychological factors includes suicidality and experiences of hospitalization for mental health [61], psychological distress by the validated Patient Health Questionnaire-4 [62] and Kessler-6 [63], gender dysphoria by a brief screening measure designed to maximize patient-centeredness [64], HRQL by the EQ-5D-5L [65], substance use and misuse by the Alcohol Use Disorders Identification Test-Concise [66] and the Drug Abuse Screening Test-10 [67], transgender integration or adaptation [68], violence victimization in childhood and adulthood [20], and stigma and discrimination by a modified version of the Everyday Discrimination Scale [69]. Structural vulnerabilities such as sex work, housing, and jail and incarceration experiences and barriers to legal gender affirmation (eg, changing name and gender marker on identification) were also measured [52,54].

### Optional HIV and STI Testing

Biological specimens are collected for HIV and/or bacterial STI testing from relevant anatomical sites of participants who have clinical indications, as determined by their medical provider. Participants for whom HIV and/or STI testing are not clinically indicated but who request and consent to the optional additional cohort testing have a flag added to their patient chart by research staff, alerting the provider to order these tests. All specimens are sent to the clinics’ site labs for analysis, per medical department procedures. Participants complete these tests in concert with their routine lab work. Test results are extracted from their patient chart.

### Study Retention

We expect to retain approximately 85% of those who consent to additional procedures (electronic survey and HIV or STI testing) across 12 months of follow-up. Data informatics personnel and research assistants at each site work collaboratively to track cohort participants and contact participants when it is time to take their follow-up surveys. Study activities are synced with routine clinical care as much as possible to minimize participant burden. Study retention and participant engagement activities are ongoing. Participants can opt-in to receive study updates in the form of a newsletter. Study updates occur via email approximately 3 times over 12 months. The email contains general updates about the study (eg, how many have enrolled to date, fun facts about the cohort, and other study milestones) and infographics with deidentified preliminary demographic and other data. The email is not sent to those who have declined to receive study updates. The purpose of the newsletter is to provide progress updates to participants and promote participant engagement, including evoking feelings of being *part of* and actively contributing to the project and the study team at each site. The newsletter emails are sent via a health insurance portability and accountability act (HIPAA)–compliant mass-messaging platform.

### LEGACY RDW Procedures

The LEGACY RDW is a HIPAA limited data set. The HIPAA limited data set may contain extensive clinical information on study participants but limits patient identifiers and other unique characteristics to preclude the possibility that the patient could be identified using data transmitted to the RDW.

### Quality Assurance

The LEGACY RDW employs various data resources to aid in the quality, maintenance, and security of patient data. Data resources are maintained, regularly monitored, evaluated, and updated. For survey data, quality assurance (QA) begins in the recruitment process. Recruitment scripts screen health record data to determine prospective patients’ eligibility before outreach. Research assistants review the outputs for these scripts and report any suspected ineligible patients so that necessary updates may be applied. Patients deemed eligible by the recruitment scripts are then offered a screener where they self-report eligibility criteria before the consent process. The survey uses restricted input options and pathing logic to promote data accuracy and completeness. In some cases, key survey data points are crosstabulated and reviewed for any erroneous response patterns not identified by the survey’s built-in QA tools. QA for the LEGACY RDW includes running scripts across data tables to check for errors, such as duplicated rows and orphaned records. The LEGACY RDW relies on the QA processes managed by the native EHR systems for measures of completeness and accuracy. Staff at both sites regularly maintain extraction, transformation, and loading scripts to ensure they are congruent with the most recent version of their native EHR systems.

### Data Sources

**Figure 2:**
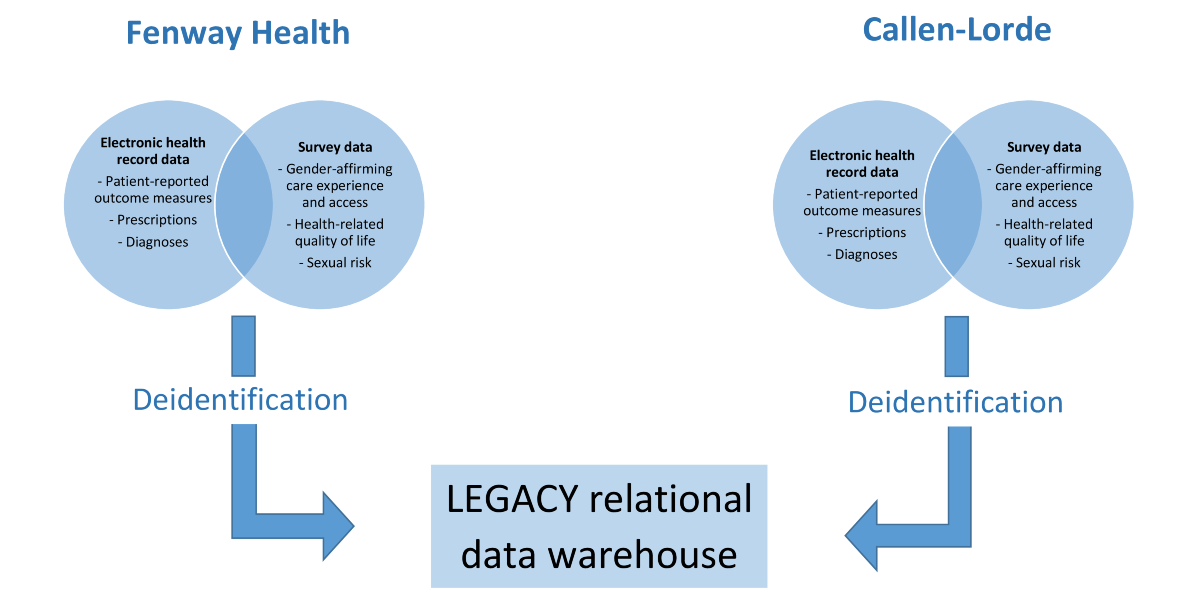
LEGACY cohort: study flow diagram. EHR: electronic health record.

The LEGACY RDW integrates outpatient, health center, and patient self-reported survey data for TGD patients into a single data management system (Figure 2). The comprehensive EHR systems at Fenway Health (Centricity Practice Solutions, athenahealth product) and Callen-Lorde (NextGen Ambulatory EHR, version 5.9/8.4) include patient demographics; registration and appointments; claims; encounter or problem list diagnoses; vital signs; lab orders and results; prescriptions; procedure or referral orders; provider notes; PROs conducted as part of routine clinical care (eg, screening for smoking, depression, and violence); imaging services; behavioral health data; dental care data; and information on patients with acute, chronic, or episodic conditions requiring special attention. These comprehensive outpatient data sources capture information on a broad range of primary care services, the most common services used by US patients. These data sources also include specialty care services to inform research questions regarding medical gender affirmation, HIV prevention, and HIV care. The LEGACY RDW is designed to enhance the breadth of the horizontal outpatient data sets by adding vertical depth with self-reported survey data from patients. Integrating electronic patient-reported survey data allows gathering of information that is not currently captured in the patient record (eg, incarceration, desires for gender-affirming procedures, and experiences in health care) to achieve the main study aims.

### EHR Data Submission Procedures

A data usage agreement (DUA) was established before Callen-Lorde shares any identifying information regarding their patients with Fenway Health.

Data come directly from the partnering site. Partners have access to a secure FTP (file transfer protocol) server within Fenway Health’s firewall to transfer the limited data set. Data partners have their own user IDs and passwords, and each data partner’s data set is segregated into individual directories. A waiver of consent and authorization specifically for patient data coming from partners’ EHRs was granted because it is not practicable to go back and obtain consent and authorization from the over 5000 patients whose health records make up the RDW.

### LEGACY Surveys

Self-report survey data for TGD patients are collected using REDCap [70,71]. REDCap is a secure web-based app validated to ensure HIPAA-compliant data collection. Survey data collected through REDCap are stored on a secure Fenway Health server before being extracted and transferred into the LEGACY RDW. Consent obtained before survey administration is stored in REDCap, with survey data and transferred into the LEGACY RDW along with survey responses.

Although the survey is hosted on Fenway Health’s servers, each research site manages survey administration for their own patients. Patient email addresses are maintained within their REDCap study records to enable the emailing of surveys to patients. To permit Callen-Lorde staff to enter their patients’ email addresses into Fenway Health’s REDCap server, both parties signed a DUA that covers sharing Callen-Lorde’s patients’ email addresses with database administrators at Fenway Health. Callen-Lorde was given their own REDCap project and log-in credentials for managing their surveys. Database administrators at Fenway created REDCap user access groups that restricted other Fenway Health staff from accessing Callen-Lorde’s REDCap records and restricted Callen-Lorde staff from accessing Fenway Health REDCap records. Fenway Health database administrators are the only staff members with access to REDCap study records for both sites.

Fenway Health uses patient medical record numbers (MRNs) as the primary identifier for their patients’ REDCap study records. Patient MRNs are replaced with LEGACY study IDs before the survey data are imported into the LEGACY RDW. Callen-Lorde preassigns LEGACY study IDs to their patients and uses the study ID as the primary identifier in their patients’ REDCap study records. Callen-Lorde’s study staff maintains their own link file connecting their patients’ study records back to their MRNs outside of REDCap. Callen-Lorde’s patients’ MRNs are never entered into the REDCap study records.

### Security and Confidentiality

The LEGACY RDW is stored within Fenway Health’s secure firewall on a server requiring log-in credentials from authorized Fenway Health staff. Only the Fenway Health database administrators assigned to the project have direct access to the full RDW. All other study personnel who require access to any data elements are given access to the appropriate data elements according to their role and need. In preliminary steps, data structures are designed to separate personal identifiers from other critical data, further enhancing protection. All partnering organizations meet or exceed the requirements for patient data safety established in the federal HIPAA guidelines.

### Confidentiality Agreements

All persons employed by Fenway Health and Callen-Lorde sign a confidentiality agreement. Fenway Health has an excellent record of using EHR data for research without breach of confidentiality. No individual-identifying data will be published or released, and data will be summarized and presented in public forums only as aggregate measures or as results from statistical analyses.

### Statistical Considerations

The study outcomes are viral suppression (for TGD patients living with HIV); PrEP uptake (for TGD patients not living with HIV); and incident syphilis, gonorrhea, and chlamydia diagnoses by anatomical site (for all patients, irrespective of HIV status). The primary exposure is medical gender affirmation (hormones and surgery).

### Sample Size

We assumed α=.05 (two-tailed; type I error rate) and β=.20 (type II error rate) for sample size estimation. The primary power analysis is based on virologic suppression (<200 copies per ml: yes or no) for TGD patients living with HIV (Tables 2 and 3). We hypothesize that medical gender affirmation (hormones and surgery vs none) will increase the proportion of HIV-infected TGD patients achieving viral suppression across follow-up. There is substantial variability in viral suppression rates among TGD people (eg, 50%-81%) [10-12,72,73]. A 25% increase in the proportion of TGD patients achieving viral suppression (moderate treatment effect), from 51% at baseline to 76% at follow-up, will require a minimum sample size of 182 patients living with HIV. Analyses of PrEP uptake and STI diagnoses are equally well-powered.

**Table 2:** LEGACY cohort: parameter estimates used in sample size estimation for viral suppression, pre-exposure prophylaxis uptake, and sexually transmitted infection diagnosis.

Outcome variable		Parameter estimate
**Viral suppression**		
	P_0_^a^	0.51
	P_1_^b^	0.76
	Odds ratio^c^	3.04
	Risk ratio^d^	1.49
**PrEP^e^ uptake**		
	P_0_	0.011
	P_1_	0.048
	Odds ratio	4.53
	Risk ratio	4.36
**STI^f^ diagnosis**		
	P_0_	0.052
	P_1_	0.210
	Odds ratio	4.85
	Risk ratio	4.04

^a^P_0_: risk in group 0 (baseline risk).

^b^P_1_: risk in group 1 (exposed).

^c^Odds ratio: ((P_1_/(1-P_1_))/(P_0_/(1-P_0_)).

^d^Risk ratio: (P_1_ to P_0_).

^e^PrEP: pre-exposure prophylaxis.

^f^STI: sexually transmitted infection.

**Table 3:** LEGACY cohort: sample size estimation for viral suppression, pre-exposure prophylaxis uptake, and sexually transmitted infection diagnosis.

Outcome variable			Outcome+		Outcome−		Total
**Viral suppression**							
	Group 1	113		36		149	
	Group 0	17		16		33	
	Total	130		52		182	
**PrEP^a^ uptake**							
	Group 1	49		967		1016	
	Group 0	2		221		223	
	Total	51		1188		1239	
**STI^b^ diagnosis**							
	Group 1	45		168		213	
	Group 0	2		45		47	
	Total	47		213		260	

^a^PrEP: pre-exposure prophylaxis.

^b^STI: sexually transmitted infection.

### Data Analysis

Descriptive statistics (eg, frequencies, means, and standard deviations) will be obtained to summarize the variables. Bivariate tests (*t* tests or *χ*^2^) will examine differences by site. Subsequent analyses will use appropriate statistical procedures to adjust for site differences if necessary. Bivariate tests (*t* tests or *χ*^2^) will examine medical gender affirmation by HIV outcomes of interest, followed by multivariable regression models. Analyses will use SAS software with two-tailed tests and an alpha .05-level of significance.

Aim 1 analyses will involve descriptive statistics to characterize medical gender affirmation exposures and HIV prevention continuum (HIVPC) and HIV care continuum (HIVCC) outcomes at baseline and each follow-up over 12 months. We will model longitudinal HIV-related outcome trajectories as a function of medical gender affirmation using generalized estimating equations [74]. Models will be adjusted for individual, interpersonal, and structural covariates and confounders. Moderators (eg, age, race, and gender identity) will be tested to identify TGD patients at the highest and lowest risk of adverse outcomes. For example, we will evaluate whether racial or ethnic self-identification (people of color vs White) is an effect modifier of hormones and viral suppression (ie, whether there is heterogeneity in treatment effects by race). Analyses will be appropriately stratified for heterogeneous treatment effects.

In aim 2, we will longitudinally model within-person changes in mental health diagnoses and response to standardized behavioral assessments from baseline (prehormones) to 12-months (aim 2) among TGD patients prospectively initiating hormone therapy at cohort entry. Mediational models will test whether changes in mental health explain the effect of medical gender affirmation on improved HIVPC or HIVCC outcomes.

Aim 3 analyses will entail descriptive statistics to characterize patient satisfaction with medical gender affirmation received, unmet needs and future desires for medical gender affirmation, and barriers and facilitators of medical gender affirmation.

Missing data can create significant problems in the analysis or interpretation of longitudinal data. Statistical summaries will be used to describe the missing data. We will assess patterns of missing data between or within follow-ups [75-77], comparing patients in care with those who drop out of care. We will use modern missing data techniques as appropriate, such as multiple imputation [78,79]. The impact of unmeasured confounders will be evaluated via sensitivity analyses [80]. We will also test for heterogeneity of treatment effects in medical gender affirmation and HIVPC or HVCC outcomes, consistent with the PCORI (Patient-Centered Outcomes Research Institute) methodology standards.

Fenway Health is the lead for data analysis and the creation of analytic data sets. This group is staffed by doctoral and master’s level biostatisticians who have extensive experience analyzing health outcomes using various statistical approaches. For LEGACY, they will be the primary resource for statistical consulting on any future grant proposals, concept sheets, study design, statistical analyses, and manuscript preparation. A delineated concept proposal process is in place to facilitate collaborations or data requests for the cohort. Any requests for access to the analysis data sets require previous approval from the principal investigator and applicable oversight bodies such as PCORI or the Fenway IRB.

## Results

### Formative Research Findings: Focus Groups

The contract began in April 2018. The Fenway Health IRB approved the formative focus group procedures in June 2018. All formative focus groups were conducted between August and October 2018. Among the 28 focus group participants, the mean age was 34 years (range 18-66 years); 13 identified as female, 12 were identified as male, and 3 identified as nonbinary; 12 were White, 5 Black or African American, 5 multiracial, 3 Asian or Pacific Islander, and 3 other race; and 8 identified as Hispanic or Latinx.

Several themes emerged from focus groups that informed cohort protocol and procedures:

1. Study population: participants strongly advocated for the inclusion of gender nonbinary patients in the research. They suggested that the team intentionally outreach to and engage TGD communities for inclusion and participation.
2. Research topics: participants felt that the reinforcement of negative transgender narratives was the main cause of research fatigue in the TGD community. Thus, they wanted the study to ask TGD patients about resiliencies and strengths, in addition to disparities and deficits.
3. Integration of clinical care and research: participants liked how the study was being integrated into their primary care, making participating efficient and low barrier. Most participants were comfortable with their medical records being accessed for the purposes of the study. Participants felt specimen collection for HIV or STIs should be optional and that the uses of the specimens should be clearly and transparently explained through an informed consent process separate from that of the survey.
4. Incentives for participation: there was a range of opinions regarding financial compensation and incentives. In 2 of the focus groups, participants strongly felt that the survey portion of the study should be remunerated, citing financial disparities facing TGD populations. Other groups felt that financial compensation was not required. These participants felt that the survey content alone would keep participants engaged and that the mission of the project was compensation enough.
5. Dissemination activities: focus group participants expressed the importance of disseminating research findings back to the community. They wanted results to be shared throughout the entire research process, rather than waiting until the end to hear about it or to not hear about it at all.

### Cohort Recruitment, Enrollment, and Retention

**Figure 3:**
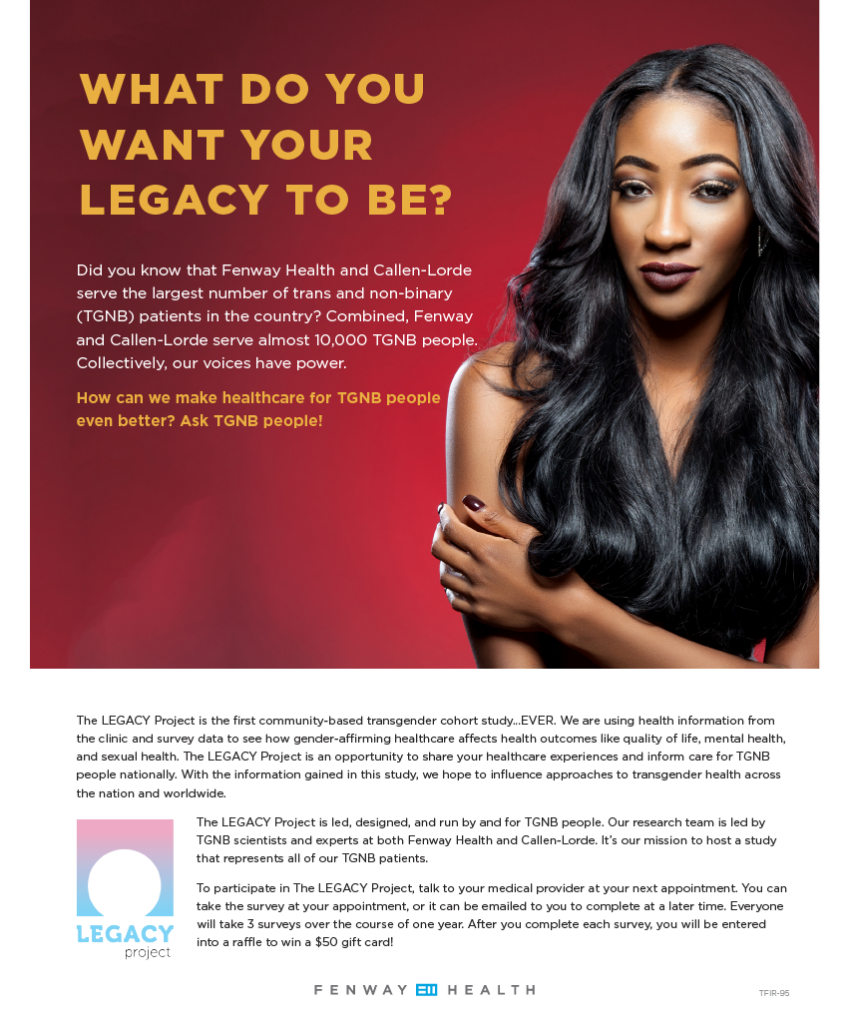
LEGACY cohort: example of study recruitment flyer.

**Figure 4:**
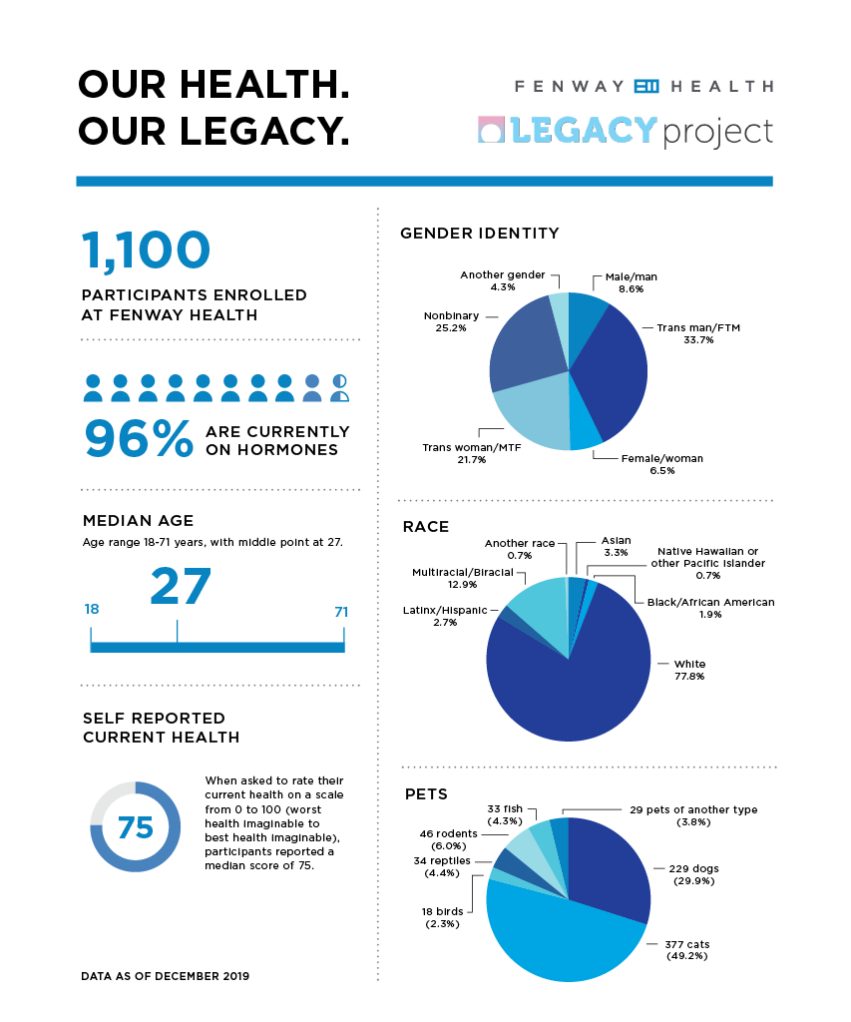
LEGACY cohort: example of participant newsletter.

The longitudinal cohort was approved by the IRB in January 2019. Prospective cohort enrollment began at Fenway Health in February 2019 and at Callen-Lorde in August 2019. Enrollment will continue through August 2020. As of April 2020, 7821 patients have been enrolled in the LEGACY RDW and 1756 have completed the additional baseline LEGACY survey. The baseline characteristics of the TGD patients in the RDW and survey are shown in Tables 4 and 5, respectively. Recruitment strategies that have demonstrated success include posting of study flyers in exam rooms and patient waiting areas (see Figure 3 for an example), educating providers about the study to facilitate successful patient referrals and linkages to the research (eg, presenting on the study to medical departments), and building a study identity that links to and is integrated with each clinical site’s transgender health program and services. Ongoing retention efforts consist of frequent reminder emails about upcoming survey participation and dissemination of study e-newsletters, which contain preliminary findings from the cohort to date (see Figure 4 for an example).

**Table 4:** Baseline electronic health record data for transgender and nonbinary adult patients (N=7821).

Sociodemographics				Values^a^, n (%)
**Age (years)**				
	18-24		2165 (27.68)	
	25-29		2117 (27.07)	
	30-39		2193 (28.04)	
	40-49		705 (9.01)	
	50-59		404 (5.17)	
	≥60		237 (3.03)	
**Gender identity**				
	Female		2900 (37.08)	
	Male		2643 (33.79)	
	Genderqueer		1700 (21.74)	
	Missing		578 (7.39)	
**Sex assigned at birth**				
	Female		4064 (51.96)	
	Male		3649 (46.66)	
	Missing		108 (1.38)	
**Race**				
	American Indian or Alaska Native		74 (0.95)	
	Asian		378 (4.83)	
	Black or African American		1070 (13.68)	
	Multiracial		475 (6.07)	
	Pacific Islander		61 (0.79)	
	White		4717 (60.31)	
	Missing		1046 (13.37)	
**Ethnicity**				
	Hispanic or Latinx		1152 (14.73)	
	Non-Hispanic or Latinx		5093 (65.12)	
	Missing		1576 (20.15)	
**Gender affirmation**				
	**Current hormone prescription**			
		Yes	6855 (87.65)	
		No	966 (12.35)	
**HIV and STIs^b^**				
	**HIV-positive serostatus**			
		Yes	421 (5.38)	
		No	7400 (94.62)	
	**Current PrEP^c^ prescription**			
		Yes	727 (9.30)	
		No	7094 (90.70)	
	**Previous STI diagnosis (non-HIV)**			
		Yes	3257 (41.64)	
		No	4564 (58.36)	

^a^Data from transgender and gender diverse patients with a primary care medical visit between January 7, 2018, and February 29, 2020.

^b^STI: sexually transmitted disease.

^c^PrEP: pre-exposure prophylaxis.

**Table 5:** Baseline patient-reported survey data for transgender and nonbinary adult patients (n=1756). Data from transgender and gender diverse patients with a primary care medical visit between January 7, 2018, and February 29, 2020.

Sociodemographics		Values, n (%)
**Age (years;** **n=1756)**		
	18-24	582 (33.14)
	25-29	440 (25.06)
	30-39	476 (27.11)
	40-71	253 (14.41)
	Missing	5 (0.28)
**Gender identity (n=1756)**		
	Trans man	743 (42.31)
	Trans woman	504 (28.70)
	Genderqueer or nonbinary AFAB^a^	382 (21.75)
	Genderqueer or nonbinary AMAB^b^	95 (5.41)
	Missing	32 (1.83)
**Sex assigned at birth (n=1756)**		
	Female	1130 (64.35)
	Male	610 (34.74)
	Missing	16 (0.91)
**Race (n=1756)^c^**		
	American Indian or Alaska Native	0 (0.0)
	Asian	52 (2.96)
	Black or African American	76 (4.33)
	Latinx	87 (4.95)
	Multiracial	245 (13.95)
	Native Hawaiian or Other Pacific Islander	11 (0.63)
	White	1253 (71.36)
	Another race	17 (0.97)
	Missing	15 (0.85)
**Ethnicity (n=1756)^c^**		
	Hispanic or Latinx	175 (9.97)
	Non-Hispanic or Latinx	1566 (89.18)
	Missing	15 (0.85)
**Sexual orientation** **(n=1756)**		
	Asexual	72 (4.10)
	Bisexual	286 (16.29)
	Gay	126 (7.18)
	Lesbian	179 (10.19)
	Pansexual	234 (13.33)
	Queer	544 (30.98)
	Questioning or unsure	54 (3.07)
	Straight or heterosexual	211 (12.01)
	Another sexual orientation	34 (1.94)
	Missing	16 (0.91)
**Educational attainment** **(n=1756)**		
	High school diploma or less	193 (10.99)
	Associate’s degree, vocational or technical school, or some college	529 (30.12)
	4-year degree	633 (36.05)
	Graduate degree	282 (16.06)
	Another level of education	17 (0.97)
	Missing	102 (5.81)
**Type of health insurance** **(n=1756)**		
	None	51 (2.91)
	Public	446 (25.40)
	Private	1182 (67.31)
	Missing	77 (4.38)
**Lifetime hormone use** **(n=1756)**		
	Taken hormones	1456 (82.91)
	Have not taken hormones but interested in taking them	234 (13.33)
	Have not taken hormones and not interested in taking them	62 (3.53)
	Missing	4 (0.23)
**Current hormone use (n=1456)**		
	Yes	1399 (96.09)
	No	57 (3.91)
**History of gender-affirming surgeries or procedures (n=1756)**		
	Yes	1009 (57.46)
	No	746 (42.48)
	Missing	1 (0.06)
**Gender-affirming surgeries or procedures by region (dichotomous; n=1009)**		
	Any facial or voice procedures	161 (15.96)
	Any chest procedures	624 (61.84)
	Any abdomen or bottom procedures	305 (30.23)
**Heard about** **pre-exposure prophylaxis** **for HIV prevention? (n=1756)**		
	Yes	1395 (79.44)
	No	240 (13.67)
	I do not know	28 (1.59)
	Missing	93 (5.30)
**Ever taken pre-exposure prophylaxis? (n=1395)**		
	Yes	136 (9.75)
	No	1255 (89.96)
	Missing	4 (0.29)
**Ever been tested for HIV? (n=1756)**		
	Yes	1151 (65.55)
	No	409 (23.29)
	I do not know	106 (6.04)
	Missing	90 (5.12)
**Result of most recent HIV test (n=1151)**		
	HIV positive	19 (1.65)
	HIV negative	1094 (95.05)
	Undetermined	7 (0.61)
	I do not know	25 (2.17)
	Missing	6 (0.52)
**Ever had an STI^d^ test (non-HIV; n=1756)**		
	Yes	1194 (68.00)
	No	372 (21.18)
	I do not know	95 (5.41)
	Missing	95 (5.41)
**Ever tested positive for an STI (non-HIV; n=1194)**		
	Yes	246 (20.60)
	No	926 (77.55)
	I do not know	15 (1.26)
	Missing	7 (0.59)
**Clinically significant depression^e^ (n=1753)**		
	Yes	604 (34.46)
	No	1149 (65.54)
**Clinically significant anxiety^e^ (n=1753)**		
	Yes	744 (42.44)
	No	1008 (57.50)
	Missing	1 (0.06)

^a^AFAB: assigned female sex at birth.

^b^AMAB: assigned male sex at birth.

^c^Race and ethnicity are assessed using a single item. Participants who select only Latinx for their racial or ethnic identity are coded to have a race of Latinx. Any participant who selects Hispanic or Latinx (not mutually exclusive) is coded to have an ethnic identity of Hispanic or Latinx.

^d^STI: sexually transmitted disease.

^e^The frequency of depressive and anxious symptoms experienced over the past 2 weeks is assessed using the Patient Health Questionnaire-4, comprising 4 items with response options ranging from not at all (0) to nearly every day (3). The cutoff for clinically significant depression is a score of >3 summed across the 2 items assessing depressive symptoms (feeling down, depressed, or hopeless and little interest or pleasure in doing things). The cutoff for clinically significant anxiety is a score of >3 summed across the 2 items for anxious symptoms (feeling nervous, anxious, or on edge and not being able to control worrying).

## Discussion

### Principal Findings

This study is an unprecedented opportunity to evaluate the impact of medical gender affirmation on HIV-related health among TGD patients, an understudied health disparities population. LEGACY will uniquely contribute to the longitudinal evidence base on medical gender affirmation and HIV-related health in TGD patients. The comprehensive research methodology links biobehavioral longitudinal data from multiple sources, including EHR, patient self-reported outcomes, and biospecimen testing. Patient-centeredness and scientific rigor are assured through the ongoing engagement of TGD people, including as community members, clinicians, scientists, and site staff.

The LEGACY cohort can serve as a platform for ongoing and new research studies. The common data model used for the study is flexible and offers the potential to easily build out and enhance the cohort with new research sites and patients in the future to create a large repository. Furthermore, the cohort infrastructure can be leveraged by other research projects, such as case-control studies interested in isolating iatrogenic effects of particular medical gender affirmation exposures, biomedical investigations such as pharmacokinetic studies of drug-hormone interactions, or mixed methods quantitative-qualitative designs to gather in-depth perspectives on health care needs. By characterizing the impact of medical gender affirmation on the lives of TGD patients, findings from the LEGACY cohort will inform evidence-based clinical care for TGD patients, including optimal interventions to improve HIV-related health disparities.

### Limitations

Limitations of the study are weaknesses inherent in a clinical cohort that recruits existing and new patients, including *clinic patient bias* and limited generalizability. For example, the cohort is relatively young in terms of age and has lower rates of lacking health insurance than previous research [24], which may challenge the generalizability of findings. Another limitation is the self-selection of participants into treatments (eg, patients self-select hormones). However, the LEGACY cohort overcomes many of the limitations of other TGD cohorts, namely, lack of racial and ethnic diversity, restriction to TGD patients in gender clinics, and a high number of nonbinary-identified patients.

### Dissemination Plans

Patients and stakeholders will be engaged in dissemination activities, both to the scientific community and the TGD communities. CAB, SAB, and RSC members will be given the opportunity to present study findings at relevant conferences and will be involved in the writing of peer-reviewed scientific manuscripts. In addition, the CAB, SAB, and RSC will be responsible for the creation and dissemination of a community report, which will outline the key study findings in lay terms and provide recommendations for community members, patients, and other key stakeholders. Patient and stakeholder partners will be involved in plans to disseminate study findings and to ensure that findings are communicated in understandable, practical, and usable ways that will inform high-quality patient-centered care for TGD people.

## Abbreviations

CAB: community advisory board
DUA: data usage agreement
EHR: electronic health record
eICF: electronic informed consent form
HIPAA: health insurance portability and accountability act
HIVCC: HIV care continuum
HIVPC: HIV prevention continuum
HRQL: health-related quality of life
IRB: institutional review board
MRN: medical record number
PrEP: pre-exposure prophylaxis
PRO: patient-reported outcome
QA: quality assurance
RDW: research data warehouse
RSC: research support coalition
SAB: scientific advisory board
STI: sexually transmitted infection
TGD: transgender and gender diverse
